# Dementia and mild cognitive impairment screening in an emergency homeless shelter

**DOI:** 10.1002/alz.13763

**Published:** 2024-03-18

**Authors:** Heather M. Ross, Primrose Dzenga, Martha Myers, Alisa Squires, Stephanie Duncan, Jamilyn Caradine, Phillip Scharf, Diana M. Bowman

**Affiliations:** ^1^ Edson College of Nursing and Health Innovation, Arizona State University Phoenix Arizona USA; ^2^ School for the Future of Innovation in Society, College of Global Futures, Arizona State University Tempe Arizona USA; ^3^ Central Arizona Shelter Services Phoenix Arizona USA; ^4^ Sandra Day O'Connor College of Law, Arizona State University Phoenix Arizona USA

**Keywords:** dementia, homelessness, mild cognitive impairment, screening

## Abstract

**INTRODUCTION:**

Older adults represent the fastest growing segment of the homeless community. Little is known about the prevalence of dementia and mild cognitive impairment (MCI) in this population.

**METHODS:**

Dementia and MCI screening using the Montreal Cognitive Assessment (MoCA) was incorporated into the standard senior evaluation for adult clients aged ≥ 55 in a large emergency homeless shelter.

**RESULTS:**

In a 6‐week period, 104 of 112 (92.9%) assessments were positive for dementia or MCI using a standard cutoff of 26, and 81 (72.3%) were positive using a conservative cutoff of 23. There was no significant difference in MoCA scores based on sex or education level, and no significant correlation between age and MoCA score.

**DISCUSSION:**

Older adults experiencing homelessness may have a high likelihood of dementia or MCI. Routine MoCA screening in older adults experiencing homelessness is feasible and can help to identify services needed to successfully exit homelessness.

## 1 BACKGROUND

Older adults comprise a rapidly growing segment of the unhoused population in the United States. Although the US Department of Housing and Urban Development Annual Homelessness Assessment Report does not specifically address older adults,^1^ census and city‐specific data indicate that approximately half of homeless adults are over age 50, with stark projected increases in the numbers and proportion of homeless adults over age 65.^2^ A 2023 cohort study of > 4000 older US adults with dementia using data from the Health and Retirement Study suggested that older adults with dementia had significant declines in wealth and increased Medicaid enrollment compared to a matched sample without dementia.^3^ The correlation of dementia to poverty, coupled with escalating rates of older adults experiencing homelessness, raises concerns that dementia and mild cognitive impairment (MCI) may be independent risk factors for homelessness in older adults. Moreover, in light of current research indicating that homelessness accelerates aging, the cognitive impacts of chronic homelessness may further exacerbate the problem for older adults.

Studies reporting prevalence of dementia and MCI in older adults experiencing homelessness have relied on prior formal diagnosis, lower sensitivity tools (e.g., Modified Mini‐Mental State [3MS]), *post mortem* examination, clinical study enrollment, or non‐shelter clinical sites.^4^, ^5^, ^6^, ^7^ Observational studies found 25% to 80% of homeless participants exhibiting some type of impairment.^5^, ^8^ Population studies of veterans experiencing homelessness found absolute risk ratio (ARR) 1.58 for dementia compared to veterans with stable housing.^9^

In a prospective study of 100 homeless adults in a single shelter setting for people experiencing homelessness using the Montreal Cognitive Assessment (MoCA) Full (https://mocacognition.com/) for dementia and MCI screening, 65% of participants screened positive for at least MCI with a standard MoCA cutoff score of ≤ 26, higher than normative population expectations. Using a more conservative cutoff of ≤ 23 to account for the cognitive stress associated with homelessness,^10^ 30% of participants screened positive.^6^

A 2022 systematic review^4^ included nine prospective studies of dementia in older homeless adults. However, the findings were noted to be limited by either selective study engagement or prior engagement with formal health‐care services related to dementia. None represented a universal screening effort. Therefore, prevalence findings were likely to be under‐representative of the true population experience. To date, there are no reported universal screening programs for dementia and MCI in older adults in emergency homeless shelter settings. We report feasibility and prevalence findings from a novel universal screening program for adults age ≥ 55 in a large, 600‐bed emergency homeless shelter using the MoCA for its established sensitivity to detect both dementia and MCI.

## 2 METHODS

Central Arizona Shelter Services (CASS), a 600‐bed emergency homeless shelter for adults in Phoenix, Arizona, conducts senior evaluation interviews for adults age ≥ 55 in the first few days after shelter intake in addition to the Vulnerability Index–Service Prioritization and Decision Assistance Tool (VI‐SPDAT) administered to all clients of homeless shelter facilities in Arizona. The senior evaluation assesses a variety of daily needs and concerns, designed to help case managers better assist older adult clients navigate social services and secure permanent housing. Motivated by observations from local public safety officials (i.e., police officers, firefighters) that older adults experiencing homelessness frequently appeared to have dementia or cognitive impairment, CASS staff added dementia/MCI screening to the senior assessment protocol.

The MoCA Full was selected due to its high sensitivity and specificity profile; reliability across multiple language, culture, and sensory needs; and validity when administered by trained non‐clinicians^11^, ^12^, ^13^ compared to other less sensitive or culturally specific screening tools (e.g., 3MS, Eight‐Item Informant Interview to Differentiate Aging and Dementia [AD8], Short Portable Mental Status Questionnaire [SPMSQ]). CASS staff and associated researchers underwent MoCA training and certification. MoCA results were incorporated into the standard case management record.

MoCA screenings were administered in a case management office immediately after the senior assessment interview when possible. When a MoCA screening was postponed due to scheduling conflicts, it was conducted on an ad hoc basis in a case management office or in a separated segment of a congregate day room in response to client preference. MoCA Full paper version 8.1 was used for all screenings, including English, Spanish, or English MoCA‐BLIND versions based on client communication needs. An evaluation study of the MoCA program implementation and outcomes was deemed exempt by the Arizona State University Institutional Review Board.

**RESEARCH IN CONTEXT**

1. **Systematic review**: The authors reviewed the literature using traditional (e.g., PubMed) sources and government reports. While there have been a few small studies of dementia in homeless older adults, there is no report of screening programs or prevalence in this population.
2. **Interpretation**: Our findings from a shelter‐based screening program suggest a very high prevalence of possible mild cognitive impairment (MCI) or dementia in older adults experiencing homelessness. This hypothesis is elevated from clinical research findings currently in the public domain.
3. **Future directions**: The feasibility and prevalence findings from this single‐site screening program should be validated across other homeless shelter sites and geographies. If validated, these findings suggest broad adoption of routine dementia and MCI screening for older adults in homeless shelter settings to assist case managers, as well as primary care and community health settings to identify older adults at risk of homelessness.

## 3 RESULTS

From September 6, 2023, to October 25, 2023, 132 CASS clients were approached for MoCA screening. One hundred twelve (83.5%) screenings were completed (Table 1). One (0.7%) client who is deaf and unable to verbalize to complete the MoCA‐HI (hearing impaired) version deferred screening until an American sign language interpreter was available. Fifteen (11.2%) clients who completed the senior evaluation prior to the initiation of the MoCA screening program did not respond to a written invitation. Four (2.9%) clients refused MoCA screening. Reasons cited for refusal included fatigue from poor sleep and concern about losing access to services including re‐housing in the event of documented memory loss or cognitive impairment.

**TABLE 1:** Montreal Cognitive Assessment screening client characteristics.

Screened client characteristics	*N* = 112 (%)
Sex	
Male	75 (67%)
Female	37 (33%)
Education attainment	
>12th grade	60 (54%)
≤12th grade	52 (46%)
Age (years)	63.2 ± 5.6

Using a standard cutoff score of 26, 104 (92.9%) of screened clients had a positive screen for at least MCI (Table 2). Using a conservative cutoff score of 23 to account for cognitive stress associated with homelessness,^6^, ^10^ 84 (72.3%) of screened clients had a positive screen for at least MCI.

**TABLE 2:** MoCA screening results.

MoCA score	*n* (%^a^) (*N* = 112)	Interpretation
30	0	Likely no dementia/MCI using standard cutoff score.
29	0	
28	3 (2.7%)	
27	5 (4.5%)	
26	4 (3.6%)	
25	9 (8.0%)	Likely no dementia/MCI using conservative cutoff score. Possible MCI using standard cutoff score.
24	10 (8.9%)	
23	9 (8.0%)	
22	8 (7.1%)	Possible dementia/MCI.
21	14 (12.5%)	
20	6 (5.4%)	
19	6 (5.4%)	
18	2 (1.8%)	
17	10 (8.9%)	
16	6 (5.4%)	
15	4 (3.6%)	
14	4 (3.6%)	
13	4 (3.6%)	
12	1 (0.9%)	
11	2 (1.8%)	
10	1 (0.9%)	
9	1 (0.9%)	
8	1 (0.9%)	
7	0	
6	0	
5	0	
4	0	
3	1 (0.9%)	
2	0	
1	0	
0	1 (0.9%)	

Abbreviations: MCI, mild cognitive impairment; MoCA, Montreal Cognitive Assessment.

^a^ Percentages do not add to 100 due to rounding.

Independent sample two‐tailed *t* tests were performed to assess differences in MoCA scores between sexes and education levels (≤ 12th grade or > 12th grade). There was no significant difference in MoCA score between male and female clients (*t*_110 _= 0.41, *P* = 0.68). There was no significant difference in MoCA score between clients with 12th grade education or less and those with more than a 12th grade education (*t*_110 _= 1.14, *P* = 0.26). There was no significant correlation between age and MoCA score (*r *= −0.142, *P* = 0.15).

A concern was raised about inconsistent screening conditions, with some screenings conducted in a semi‐private area of a congregate dayroom rather than in a private case management office. Of 18 screenings performed in a dayroom as opposed to a case management office, 15 (83%) were positive using the standard cutoff of 26, and 9 (50%) were positive using the conservative cutoff of 23. These findings suggest no deleterious effect of screening in a potentially distractible location compared to screening in a private setting without potential distraction.

## 4 DISCUSSION

Unhoused older adults in an emergency homeless shelter largely accept MoCA screening for dementia or MCI as part of the case management process, and universal MoCA screening is feasible for shelter case management staff to conduct. Universal screening of older adult clients in a homeless shelter setting reveals a higher rate of positivity for possible dementia or MCI (92.9% using a standard cutoff score of 26, or 72.3% using a more conservative cutoff score of 23) than the reported literature from shelter‐based screening interventions, which relied on prior dementia diagnosis or prospective study enrollment rather than universal screening using a tool with high sensitivity for dementia and MCI. Similarly, the findings from this universal screening program reveals higher positivity than screening programs conducted in health‐care settings, suggesting that unhoused older adults with dementia or MCI may not access health care and other supportive services including cognitive screening at the same rate as unhoused older adults without cognitive impairment.

The high prevalence of positive screens for MCI or dementia in older adults experiencing homelessness highlights the value of providing screening in health care and other service settings for vulnerable populations to identify service needs to break the homelessness cycle and prevent return to homelessness. Screening for dementia and MCI as a functional assessment across a variety of settings is particularly important to support re‐housing efforts in light of calls to use return to homelessness as a key performance indicator for shelter and other social service institutions,^14^ as current assessment tools used in homeless shelter settings (e.g., VI‐SPDAT) rely on self‐report of non‐specific vulnerabilities rather than reliable screening tools. People who are subject to one or more social or structural vulnerabilities may not access primary care services including preventative screenings as a routine health maintenance practice for a variety of reasons. Therefore, in addition to incorporating routine dementia/MCI screening for all older adults in primary care settings, screening in non‐primary care health settings (e.g., urgent care) and non–health‐care settings may reach additional segments of the population to trigger further evaluation and intervention in advance of becoming unhoused. Universal screening both in health‐care and non–health‐care settings merits further study for feasibility, acceptability, and impact in preventing homelessness and other undesired sequelae associated with dementia/MCI in older adults.

The findings from universal MoCA screening in an emergency homeless shelter environment additionally highlight the need for pathways to establish follow‐up diagnostic and management care for homeless older adults with previously undiagnosed dementia or MCI. Integration of social and health services for vulnerable populations, including engagement of courts for conservatorship considerations, may enable more successful exit from homelessness by connecting clients to appropriate supportive services when they may lack the cognitive insight to fully understand their needs.

Even without direct health‐care service provision, dementia/MCI screening provides valuable information for homeless shelter professionals to better assist clients to successfully exit shelter services to permanent stable housing. Shelter staff, including case managers, reported that MoCA screening results enhanced their understanding of client needs, in turn allowing them to better manage and guide older adult clients to supportive services. In at least one case, case managers were able to notify emergency medical staff about a client's MoCA screening, which led to formal evaluation and diagnosis in a hospital setting, and ultimately resulted in the client being able to access long‐term care placement for dementia. Future work is needed to correlate MoCA findings with client‐reported mental health needs on the VI‐SPDAT intake, as well as prospective use of subsection category scores to guide future housing placements. Furthermore, the findings from this screening program highlight the need for additional housing options to prevent return to homelessness for older adults with cognitive deficits.^14^

Universal screening programs may help to describe the true prevalence of dementia/MCI in older adults as well as the social risks associated with dementia/MCI and association of dementia/MCI and length of stay in shelter or chronic homelessness. Future work is needed to establish whether dementia/MCI may be an independent risk factor for homelessness in the United States. A clearer understanding of dementia/MCI prevalence and risk for homelessness may motivate and inform the development of upstream policy and social programs to prevent older adults from becoming unhoused. Comparative studies in international contexts may further illuminate policy opportunities for prevention and response to homelessness in older adults for whom dementia/MCI may be a contributing risk factor.

### 4.1 Limitations

These findings are limited by a single‐site setting of older adults in an emergency shelter in a single metropolitan area in the United States. Future work is needed to validate these findings with other unhoused older adult populations including unsheltered individuals, individuals in other rural and urban regions in the United States, and individuals in other countries with different social policy structures informing health, housing, and aging structures.

## CONFLICT OF INTEREST STATEMENT

The authors declare no conflicts of interest. Author disclosures are available in the supporting information.

## CONSENT STATEMENT

The study was granted exemption by the Arizona State University Institutional Review Board.

## Supporting information

Supporting Information
